# Unlocking the potential of flavonoid biosynthesis through integrated metabolic engineering

**DOI:** 10.3389/fpls.2025.1597007

**Published:** 2025-05-29

**Authors:** Yuan Wang, Jiahong Chen, Genhe He, Li Yin, Yonghui Liao

**Affiliations:** ^1^ Key Laboratory of Jiangxi Province for Functional Biology and Pollution Control in Red Soil Regions, School of Life Sciences, Jinggangshan University, Ji’an, China; ^2^ Key Laboratory of Grain Crop Genetic Resources Evaluation and Utilization, Ministry of Agriculture and Rural Affairs, Shanghai Agrobiological Gene Center, Shanghai, China

**Keywords:** flavonoid biosynthesis, transcriptional regulation, plant cell factory, metabolic engineering, artificial intelligence

## Abstract

Flavonoids are a diverse class of plant polyphenols with essential roles in development, defense, and environmental adaptation, as well as significant applications in medicine, nutrition, and cosmetics. However, their naturally low abundance in plant tissues poses a major barrier to large-scale utilization. This review provides a comprehensive and forward-looking synthesis of flavonoid biosynthesis, regulation, transport, and yield enhancement strategies. We highlight key advances in understanding transcriptional and epigenetic control of flavonoid pathways, focusing on the roles of MYB, bHLH, and WD40 transcription factors and chromatin modifications. We also examine flavonoid transport mechanisms at cellular and tissue levels, supported by emerging spatial metabolomics data. Distinct from conventional reviews, this review explores how plant cell factories, genome editing, environmental optimization, and artificial intelligence (AI)-driven metabolic engineering can be integrated to boost flavonoid production. By bridging foundational plant science with synthetic biology and digital tools, this review outlines a novel roadmap for sustainable, high-yield flavonoid production with broad relevance to both research and industry.

## 1 Introduction

Flavonoids are a widespread class of polyphenolic compounds found across the plant organs, including fruits, vegetables, grains, bark, roots, stems, and flowers (Chandra et al., 2016). Synthesized through the phenylpropanoid pathway, these compounds are celebrated for their health-promoting properties, making them valuable in industries like nutraceuticals, pharmaceuticals, and cosmetics (Roy et al., 2022). Their benefits include antioxidative, anti-inflammatory, anti-mutagenic, and anti-carcinogenic properties, as well as the ability to modulate key cellular enzyme functions (Chandra et al., 2016; Ekalu and Habila, 2020); Flavonoids have demonstrated capacities in free radical scavenging, prevention of coronary heart disease, hepatoprotective, anti-inflammation, and anticancer activities (Kumar and Pandey, 2013 2013). More recently, research has uncovered their potential as antiviral agents, adding another layer to their therapeutic promise (Zhang et al., 2023).

In plants, flavonoids are indispensable. They help fend off oxidative stress, regulate growth, and contribute to processes like auxin transport, root and shoot development, pollination, and the management of reactive oxygen species (ROS). They’re also key players in the signaling that underpins the symbiotic relationship between legumes and nitrogen-fixing *Rhizobium* bacteria (Weston and Mathesius, 2013). Beyond this, flavonoids shape how plants interact with their surroundings—whether with microbes, animals, or other plants—and bolster resilience against environmental challenges (Mierziak et al., 2014). Recent work has shown they can enhance soil chemistry in the rhizosphere, improve nutrient uptake, and even attract beneficial microbial communities, underscoring their ecological significance (Wang et al., 2022). Flavonoids prevent ROS generation by suppressing singlet oxygen, inhibiting ROS-generating enzymes such as cyclooxygenase, lipoxygenase, monooxygenase, and xanthine oxidase, chelating transition metal ions such as Fe^2+^ and Cu^2+^, and recycling other antioxidants (Baskar et al., 2018).

Flavonoids are associated with a decreased risk of chronic diseases such as cardiovascular disease and have been linked to improved cognitive function. Research indicates that flavonoids can reduce the risk of cardiovascular disease, type II diabetes, and cancer by inhibiting the activation of certain proteins, specifically protein kinases and phospholipid kinases. This inhibition disrupts their normal function, leading to reduced oxidation, inflammation, gene mutations, and cancer development. The wide range of beneficial properties of flavonoids has led to their use in various industries, including food, pharmaceutical, and nutraceutical sectors. In the food industry, flavonoids serve as preservatives, pigments, and antioxidants. In the pharmaceutical industry, their anti-aging, antioxidant, anti-inflammatory, and anticancer properties make them valuable ingredients in numerous products (Dias et al., 2021). However, high purity and quality are essential for the industrial application of flavonoids (Addi et al., 2022). Despite their vast potential for industrial application, flavonoids face challenges that necessitate the development of strategies to improve bioavailability, create sustainable extraction and refinement technologies, and establish stability procedures to broaden their applicability (Albuquerque et al., 2021).

## 2 Structure, diversity, and biosynthesis of flavonoids

Flavonoids represent a diverse group of plant secondary metabolites characterized by a remarkable variety of chemical structures. At their core, these compounds feature a 15-carbon skeleton, typically denoted as C6-C3-C6, which consists of two phenyl rings (labeled A and B) linked by a heterocyclic ring (C) containing an oxygen atom (Safe et al., 2021). This foundational structure undergoes extensive modifications, giving rise to the vast diversity observed within the flavonoid family. Variations in unsaturation, the attachment position of the B ring to the C ring, levels of hydroxylation and oxidation, glycosylation patterns, and additional substitutions account for the structural complexity of these compounds (Dias et al., 2021). As a result, flavonoids are classified into several subclasses, including flavanones, flavonols, flavones, anthocyanidins, isoflavones, chalcones, aurones, phlobaphenes, dihydroflavonols, leucoanthocyanidins, and proanthocyanidins (Liu et al., 2021). To date, approximately 10,000 distinct flavonoid compounds have been identified in plants, underscoring their evolutionary and ecological importance (Dixon and Pasinetti, 2010).

The biosynthesis of flavonoids originates from the amino acid phenylalanine, which is converted into cinnamic acid by the enzyme phenylalanine ammonia-lyase (PAL), a cornerstone of the phenylpropanoid pathway (Davies et al., 2024). Next, cinnamic acid is hydroxylated to 4-coumaric acid by cinnamate 4-hydroxylase (C4H), a cytochrome P450 enzyme. This is followed by activation to 4-coumaroyl-CoA via 4-coumarate: CoA ligase (4CL). The resulting 4-coumaroyl-CoA combines with three malonyl-CoA molecules through the action of chalcone synthase (CHS) to produce chalcone, a molecule with two phenyl rings. Chalcone is then rapidly isomerized into flavanone by chalcone isomerase (CHI), setting the stage for further diversification (Li et al., 2021). From flavanone, the pathway splits into multiple branches. Flavanone 3-hydroxylase (F3H) converts flavanone into dihydroflavonol, which can then be directed toward flavonols by flavonol synthase (FLS) or flavones by flavone synthase (FNS), contributing to the diversity of flavonoids. For anthocyanin synthesis, dihydroflavonol is reduced to leucoanthocyanidin by dihydroflavonol 4-reductase (DFR) and subsequently oxidized to anthocyanidin by anthocyanidin synthase (ANS). The final step involves glycosylation, typically catalyzed by UDP-glycosyltransferases, which stabilizes anthocyanidin into anthocyanin—the pigment responsible for the red, purple, and blue colors in plants (Liu et al., 2021). Understanding the flavonoid biosynthetic pathway provides a foundation for metabolic engineering, allowing researchers to manipulate key enzymes or transcriptional regulators to enhance the production of targeted flavonoid compounds. This holds immense potential not only for improving plant stress resistance and crop quality but also for producing high-value bioactive molecules for pharmaceutical and nutraceutical applications (Davies et al., 2024). However, the intricate regulation of this pathway poses challenges, requiring further investigation to fully harness its potential.

## 3 Deciphering flavonoid transport at cellular and tissue-level perspective

**Figure 1:**
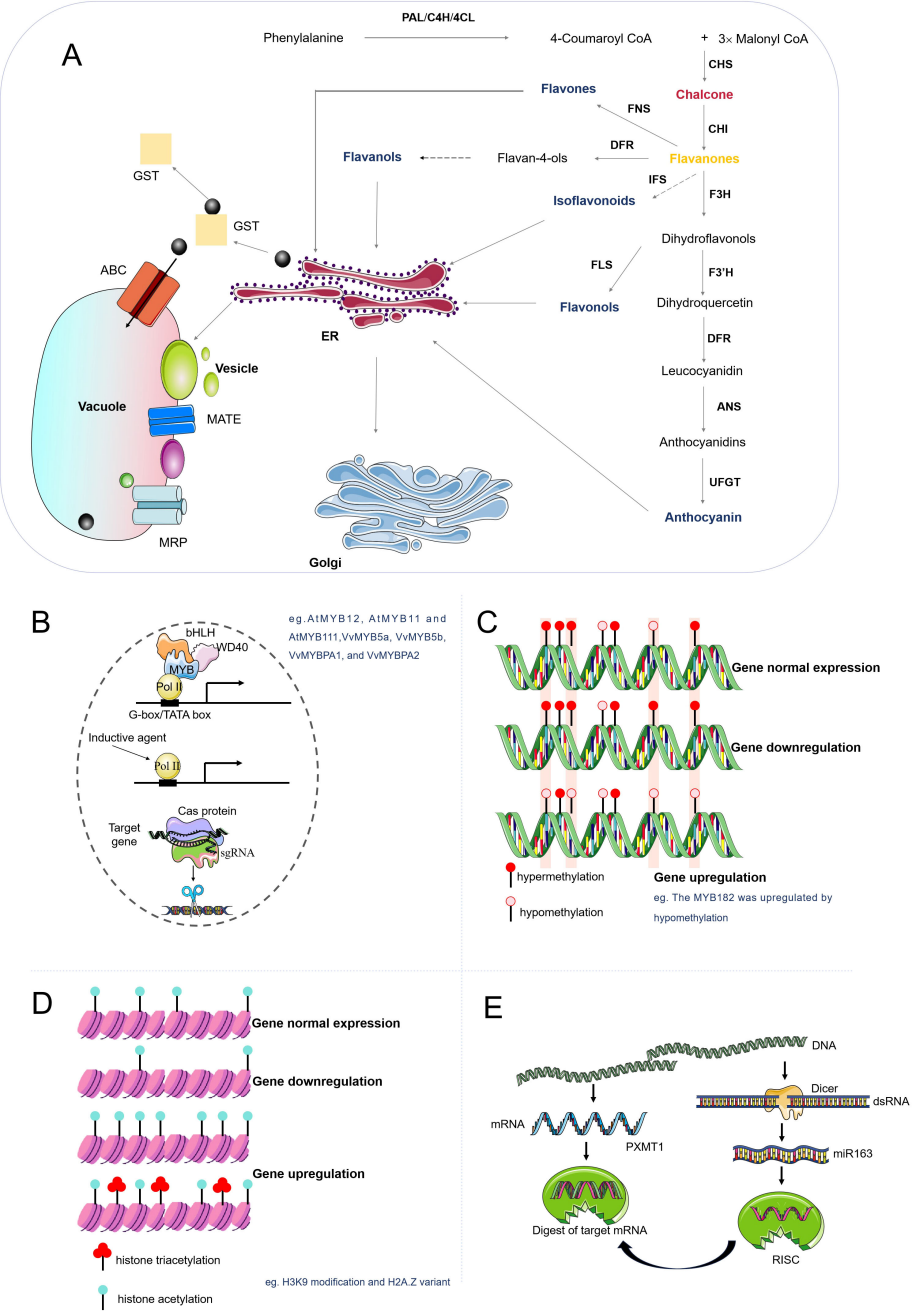
Biosynthesis, transport, and regulation of flavonoid compounds. **(A)** Flavonoids, derived from phenylpropane, are synthesized by various enzymes and transported to the vacuole via the endoplasmic reticulum or Golgi apparatus. **(B)** Other regulatory methods include the MWB complex binding to target genes, direct regulation by inducers, and gene editing technology for knockout, knock-in, and fine-tuning of gene expression. **(C)** DNA methylation influences flavonoid synthesis by regulating gene expression. Increased methylation inhibits, while demethylation enhances gene expression. **(D)** Histone modification, including acetylation and methylation, can also regulate gene expression. **(E)** siRNA, formed by Dicer cutting double-stranded RNA, binds to the AGO protein and degrades the mRNA of the target gene, thus reducing gene expression.

Synthesized through cytosolic biosynthesis, flavonoids undergo precisely regulated trafficking to their functional sites, including vacuoles, cell walls, and the apoplast (extracellular space), via diverse transport mechanisms. These processes span cellular compartments and intercellular spaces, and enable flavonoids to execute critical roles in anthocyanin-mediated pigmentation, UV-B radiation shielding, ROS scavenging, phytohormone signaling, and biotic/abiotic stress resistance (Manzoor et al., 2023). This transport involves a network of membrane-bound transporters, vesicle trafficking systems, and conjugation processes. At the cellular level, four distinct cellular transport mechanisms have been identified for flavonoid compartmentalization in plants (**Figure 1A**): (1) ATP-binding cassette (ABC) transporters, particularly multidrug resistance-associated proteins (MRPs), facilitate ATP-dependent vacuolar sequestration of flavonoid glycosides including anthocyanins. (2) Multidrug and toxic compound extrusion (MATE) transporters employ proton gradients to mediate flavonoid translocation into vacuoles or apoplastic spaces, exemplified by the *Arabidopsis* TT12 transporter functioning as a vacuolar flavonoid/H⁺ antiporter in seed coat cells accumulating proanthocyanidins (Marinova et al., 2007). (3) Glutathione S-transferase (GST)-dependent mechanisms, where specific isoforms like TT19 act as molecular chaperones to facilitate anthocyanin transport to the tonoplast through ligand binding (Sun et al., 2012). And (4) Vesicle-mediated trafficking involving endoplasmic reticulum-derived vesicles and Golgi apparatus, with potential participation of anthocyanic vacuolar inclusions (AVIs) in flavonoid deposition (Poustka et al., 2007). Emerging evidence suggests coordinated regulation by soluble NSF attachment protein receptor (SNARE) proteins and endomembrane trafficking systems, revealing intricate interactions between membrane fusion machinery and specialized metabolite storage processes (Hassani et al., 2023).

Flavonoids demonstrate systemic mobility across plant tissues through coordinated vascular transport systems (Chen et al., 2023; Dwivedi et al., 2017). These secondary metabolites are primarily synthesized in source tissues, such as leaves, and subsequently travel through the phloem to reach sink tissues, including roots, flowers, and fruits. Within source tissues, flavonoid glycosides actively load into phloem sieve tubes, a process that requires intricate coordination between mesophyll cells and companion cells. Flavonoids traverse the phloem, moving both upward (from leaves to other organs) and downward (from leaves to roots) (Buer et al., 2007). Xylem-mediated transport, conversely, occurs through passive apoplastic diffusion into vascular conduits. Root systems utilize xylem-transported flavonoids for developmental signaling and stress adaptation. Meanwhile foliar-derived compounds descending through xylem networks modulate root architecture and nutrient acquisition (Nicolas-Espinosa et al., 2023). The vascular bundle organization permits flavonoid redistribution through both symplastic (plasmodesmatal) and apoplastic transport routes, with pathway selection influenced by tissue-specific demands. Notably, root exudation of specific flavonoid derivatives into the rhizosphere mediates plant-microbe interactions and biogeochemical cycling (Hassan and Mathesius, 2012). Recent methodological advances in metabolomics have revolutionized the study of flavonoid biosynthesis dynamics. While conventional metabolomic approaches document bulk metabolite fluctuations, they lack spatial resolution to map phytochemical distributions at cellular or tissue levels. This limitation potentially obscures critical metabolic events occurring in discrete anatomical regions during developmental transitions or environmental challenges. Emerging spatial metabolomics platforms, particularly mass spectrometry imaging (MSI), enable precise visualization of flavonoid localization patterns within plant microstructures (Chen et al., 2024). This technique provides multidimensional data linking metabolite distribution with cellular ultrastructure, offering unprecedented insights into compartment-specific metabolic regulation and biochemical functionality.

## 4 Regulation of flavonoid synthesis at the molecular level

### 4.1 The role of transcription factors in the regulation of flavonoid biosynthesis

Transcription factors (TFs) are proteins that attach to specific DNA sequences and play a key role in regulating the transfer of genetic information from DNA to mRNA. Their role is particularly significant in the regulation of flavonoid biosynthesis, and they can stimulate or inhibit the expression of structural genes in biosynthetic pathways (**Figure 1B**). Various transcription factor families, including MYB, bHLH, and WD40, have been recognized as central regulators of flavonoid biosynthesis. These transcription factors form a complex, known as the MBW complex, which is crucial for the activation of late biosynthetic genes (LBGs) in the flavonoid pathway (**Figure 1B**) (Zhao et al., 2013).

MYB transcription factors are acknowledged as one of the most extensive families in the plant kingdom. These TFs are characterized by a highly conserved MYB domain located at their N-terminus. According to the number of MYB domain, MYB TFs can be divided into 1R-MYB, 2R-MYB, 3R-MYB and 4R-MYB. A specific subgroup within this family, referred to as the R2R3 MYB transcription factors, is exclusive to plant species and play a vital role in controlling the expression of structural genes. These specific genes are crucial for the biosynthesis pathways of anthocyanins, flavonoids, and monolignols (Zhao et al., 2013). In *Arabidopsis*, *AtFLS1*, which encods a flavonol synthase that facilitates the conversion of dihydroflavonols into flavonols, is regulated in a tissue-specific and developmentally controlled manner by three R2R3-MYB proteins: MYB12, MYB11, and MYB111 (Schilbert and Glover, 2022). Similarly, in grape, the flavonoid pathway is regulated by at least four MYB TFs: VvMYB5a, VvMYB5b, VvMYBPA1, and VvMYBPA2 (Liang et al., 2022). Recent studies have shown that the transcription factor, VvMYB24, plays a central role in controlling the metabolism of terpenes and flavonoids (Nguyen, 2020). In *Lycium ruthenicum*, overexpressing *LrMYB94* and *LrWRKY32* led to a significant increase in the levels of Kaempferol-3-O-rutinoside (K3R) and rutin, thereby enhancing the plant’s medicinal properties. In contrast, silencing these genes effectively impeded the light-induced synthesis of K3R and rutin, resulting in a decrease in the medicinal activity of black wolfberry (Du et al., 2024).

The bHLH (Basic helix-loop-helix) TFs also play a significant role in the biosynthesis of flavonoids. The bHLH TFs are key in managing various stages of the flavonoid pathway, including the production of anthocyanins and proanthocyanidins (PAs), underscoring their essential role in this process (Fresquet-Corrales et al., 2017). In *Arabidopsis*, the bHLH transcription factor TT8 (Transparent Testa 8) works in conjunction with the MYB transcription factors PAP1 (Production of Anthocyanin Pigment 1) and PAP2 to supervise the biosynthesis of anthocyanins (Li et al., 2020). Similarly, in *Vitis vinifera*, VvbHLH1 interacts with VvMYBPA1, playing a crucial role in the regulation of proanthocyanidin biosynthesis (Koyama et al., 2014).

WD40 proteins, distinguished by the presence of WD40 repeats, are instrumental in enabling protein-protein interactions. Within the context of flavonoid biosynthesis, these proteins serve as a scaffold for the formation of the MBW complex. For example, in *Camellia sinensis*, CsWD40 plays a significant role in the joint regulation of the flavonoid pathway (Liu et al., 2018).

Beyond the well-known MYB, MYC (also referred to as bHLH), and WD40 proteins, there exist other transcription factors that contribute to the regulation of flavonoid biosynthesis. For example, AP2/ERF and WRKY directly influence the primary flavonol synthase gene or other initial genes involved in the flavonoid biosynthesis process (Cao et al., 2024). In the case of *Artemisia annua*, the YABBY5 transcription factor is responsible for the activation of several genes in the flavonoid pathway, such as *AaPAL*, *AaCHS*, *AaCHI*, and *AaUFGT*. This activation promotes a notable rise in the total accumulation of flavonoids and an increase in anthocyanin production, which is evidenced by the deep purple coloration of the stem (Kayani et al., 2021).

Above mentioned MBW complex majorly depends on the binding to specific DNA sequence and transcription activation activity of R2R3 MYB TF. However, different MYB transcription factors may play opposite roles in flavonoid synthesis. Recent studies show that R3 and R2R3-MYB TFs can inhibit the biosynthesis of flavonoids. For example, more than 30 MYB TFs in over 15 species have been reported to inhibit flavonoid biosynthesis (Chen et al., 2019). According to the C-terminal structure of the repressive MYB TFs, their inhibition of flavonoid synthesis can be achieved in the following two modes: Some MYB transcriptional repressors do not have a transcriptional activation domain, but they compete with MYB transcriptional activators to bind to bHLH and WD40 proteins, thereby repress the expression of target genes. Other MYB transcriptional repressors contain a transcriptional repressor domain at the C-terminus, which binds to the promoter region of the target gene and directly represses its expression (Chen et al., 2019).

Furthermore, we systematically summarized both the transcription factors mentioned in this study and those not previously discussed in a tabular format (**Table 1**). Detailed descriptions of the specific flavonoid metabolic networks regulated by these transcription factors were also provided (An et al., 2019; Grunewald et al., 2012; Hou et al., 2024; Wang et al., 2023). This comprehensive presentation aims to offer readers a clearer understanding of the crucial regulatory roles that transcription factors play in the biosynthesis and metabolic processes of flavonoids in plants.

**Table 1:** Transcription factors (TFs) that involved in Flavonoid biosynthesis.

TF family	Example TF(s)	Role/function in flavonoid biosynthesis
R2R3-MYB	AtMYB11, AtMYB12, AtMYB111 (flavonol‐specific activators); AtMYB75 (PAP1), AtMYB90, AtMYB113/114 (activators of anthocyanin/proanthocyanidin genes)	Bind directly to promoters of biosynthetic genes. Generally, “MYB-B” group members (e.g., MYB11/12/111) activate early genes (such as CHS, CHI, F3H, and FLS) while “MYB-C” group TFs (e.g., MYB75/PAP1) form a complex with bHLH and WD40 to regulate later steps (DFR, LDOX, UFGT)
bHLH	TT8, GL3, EGL3 (*Arabidopsis* regulators)	Interact with R2R3‐MYBs to form the MBW complex needed for robust activation of LBGs (e.g., in anthocyanin biosynthesis
bZIP	VqbZIPC22 (from grapevine)	Regulates key downstream genes such as CHI and FLS, thereby affecting flavonol levels (e.g., kaempferol and quercetin accumulation)
YABBY	AaYABBY5 (from *Artemisia annua*)	Directly activates promoters of early (PAL, CHS, CHI) and late (UFGT) biosynthetic genes to boost total flavonoid—and in particular anthocyanin—levels
NF-Y	NF-Y subunits (e.g., NF-YA, NF-YB, NF-YC as a complex)	Regulate flavonoid biosynthesis by binding to CCAAT motifs in promoters (e.g., of CHS1) and modulating chromatin structure for transcriptional activation
AP2/ERF	CitERF32, CitERF33, CitRAV1 (from citrus)	Activate flavonoid pathway genes (for instance, CitCHIL1) thereby increasing overall flavonoid content; these factors integrate developmental and environmental signals
WRKY	AtWRKY23, AtWRKY75 (in Arabidopsis)	Implicated in modulating flavonol biosynthesis (and possibly other branches) as well as linking stress/defense signals with secondary metabolism
NAC	MdNAC52 (apple), SlNAC1 (tomato)	Enhance anthocyanin levels by modulating expression of key MYB regulators or directly influencing structural genes; integrate stress and hormonal signals
BBX (B-box)	MdBBX20, MdBBX22, AtBBX21/24	Influence anthocyanin accumulation in response to light conditions by interacting with HY5 and MYBs

### 4.2 The role of epigenetics in plant flavonoid biosynthesis

Epigenetics plays a significant role in regulating plant secondary metabolism, particularly in medicinal plants. Epigenetic modifications, such as DNA methylation and histone modifications, are central to the regulation of gene expression. These changes significantly impact a broad range of biological processes, including flavonoid metabolism. DNA methylation, a well-studied epigenetic mechanism, can induce heritable and phenotypic alterations in functional genes without changing the DNA sequence itself. Alterations in DNA methylation patterns can dictate various aspects of plant life, such as morphogenesis, growth, development, and secondary metabolite production (Kumar and Mohapatra, 2021; Liu et al., 2020). In *Arabidopsis thaliana*, DNA methylation at the promoter region of the FLS1 gene, which encodes flavonol synthase, leads to reduced expression of this gene and consequently lower flavonol content (Zhao et al., 2023). MYB182, a repressor of anthocyanin synthesis in poplar, becomes hypomethylated under dark conditions, resulting in elevated MYB182 expression (**Figure 1C**). Consequently, downstream genes related to anthocyanin synthesis are inhibited, leading to reduced anthocyanin content (Fan et al., 2018). Histone modifications, including acetylation and methylation, also play essential roles in gene expression regulation (**Figure 1D**). For example, acetylation at histone H3K9 has been observed to modulate the gene expression of key enzymes in the flavonoid and abscisic acid pathways, thereby enhancing the drought resistance of sea buckthorn (*Hippophae rhamnoides*) (Li et al., 2023). In *Petunia hybrida*, histone acetylation at the promoter region of the CHS gene (encoding chalcone synthase) enhances its expression, thereby increasing chalcone production (Liu et al., 2021). Additionally, the histone variant H2A.Z has been implicated in various plant physiological programs, including flavonoid biosynthesis (Nguyen, 2020). In *Matthiola incana*, histone methylation at the DFR gene, which encodes dihydroflavonol reductase, results in higher expression levels and increased anthocyanin accumulation (Winkel-Shirley, 2001). MicroRNAs (miRNAs), which are negative regulators of gene expression, bind to target gene sequences, reducing their expression (**Figure 1E**). In *Arabidopsis*, miR163 regulates flavonoid synthesis by targeting the PXMT1 gene (Fan et al., 2018). Collectively, these findings underscore the intricate relationship between epigenetic modifications and plant metabolism, emphasizing the need for further research in this captivating field.

## 5 Strategies to improve flavonoid contents in plants

### 5.1 Unleashing the power of genetic engineering in flavonoid biosynthesis of plant

Genetic engineering has ushered in a new era in plant research, particularly in enhancing flavonoid production. This advancement is primarily achieved through plant transgene and gene editing techniques (**Figure 1**). However, the intricate and diverse metabolic pathways involved in flavonoid biosynthesis pose a significant challenge in obtaining high yields of specific flavonoids. Synthetic biology approaches, such as the utilization of transcription factors and enzyme diversity, have shown promise in enhancing flavonoid yields and broadening their production spectrum. For instance, the overexpression of the PAP1 transcription factor in *Arabidopsis* leads to alterations in the plant’s metabolic profile, including a surge in flavonoid production (Fu et al., 2021; Mitsunami et al., 2014). Moreover, a novel biotechnology for in-planta gene editing was employed to promote flavonoid biosynthesis. Consequently, this leads to an increased flavonoid content in bamboo leaves by knocking out the *Cinnamoyl-CoA Reductase* (*CCR*) gene (Sun et al., 2023). Zeng et al. (2021) used the CRISPR/Cas9 tool to successfully engineer high-yield hyoscyamine production from Belladonna plants for the first time. This achievement underscores the potential of genetic engineering in enhancing flavonoid production (**Figure 1B**). Collectively, these studies underscore the transformative potential of genetic engineering in advancing flavonoid biosynthesis pathways within plant systems.

### 5.2 Plant cell factories: a sustainable approach to flavonoid production with the industrialization of plant cell cultures

Traditional flavonoid production methods, including chemical synthesis and biological extraction, are limited by low yields, environmental variability, and high costs (Chaves et al., 2020; Sheng et al., 2020). Plant cell culture technologies have emerged as viable alternatives for the production of bioactive compounds, and offer the added benefit of preserving local ecosystems. These technologies enable the cultivation of plant cell suspensions, which allows for the production of valuable molecules independent of environmental, geographical, and seasonal constraints. Additionally, this method reduces production costs, improves product safety, and enables scalable manufacturing (Verpoorte et al., 2000). The *in vitro* cultivation of plant cell cultures is frequently complicated by multiple technical challenges throughout the experimental workflow. Principal limitations include slow growth rates, metabolic constraints, microbial contamination, cost of infrastructure, and genetic instability (**Table 2**).

**Table 2:** The advantages and challenges of plant cell factories.

Advantages of the approach	
Environmental Independence	Production is decoupled from external factors like climate or soil quality, enabling year-round operation
Sustainability	By minimizing the need for extensive agricultural land and reducing pressure on wild plant populations, this method aligns with conservation goals
Scalability	Bioreactor-based systems can be scaled from laboratory to industrial levels, accommodating market demand with relative ease
Product Consistency	Cultured cells synthesize flavonoids with biochemical profiles identical to those of intact plants, preserving their therapeutic efficacy
Challenges in implementation	
Slow Growth Rates	Plant cells proliferate more slowly than microbial systems, extending production timelines and increasing operational costs
Metabolic Constraints	Flavonoid biosynthesis is tightly regulated, often resulting in suboptimal yields without genetic or environmental intervention
​Microbial contamination	bacterial, fungal, or endogenous pathogens contamination
Cost of Infrastructure	Bioreactors, specialized media, and downstream processing equipment require significant investment
Genetic Stability	Prolonged subculturing can lead to somaclonal variation, potentially reducing metabolite consistency

**Figure 2:**
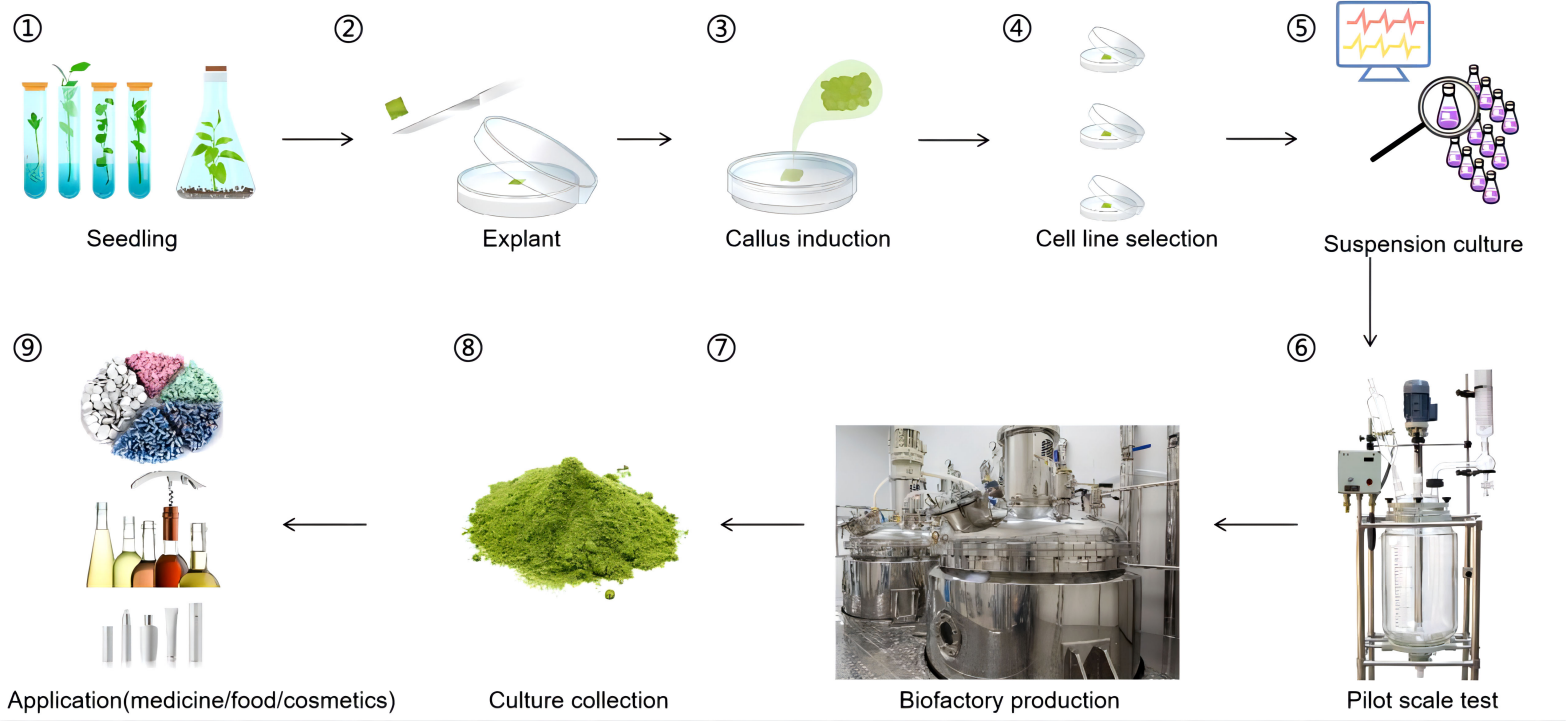
Production of flavonoids using a plant cell factory. ① Seedling growth: Sterile seeds are planted in a culture medium to grow and obtain sterile seedlings. ② Tissue selection: Suitable tissues are selected from the sterile seedlings to serve as explants. ③ Callus induction: The explants are induced appropriately to form callus tissue. ④ Cell line selection: From a large amount of callus tissue, cell lines that can produce the target compound in large quantities are selected. ⑤ Suspension culture: The selected cell line is cultured in suspension to determine the optimal growth conditions for the cell line. ⑥ Scale-up validation: Pilot-scale bioreactor runs (10 L) confirm stable flavonoid productivity. ⑦ Large-Scale production: The target cell line is produced in large quantities using a fermenter to obtain a large amount of the target compound. ⑧ Separation and purification: The products of the cells after fermentation are separated and purified. ⑨ Commercialization: The obtained target product can finally be applied in medicine, food, and cosmetics.

A notable example is *Hypericum perforatum* (St. John’s Wort), a medicinal plant renowned for its antidepressant properties and high flavonoid content. Researchers have successfully established cell suspension cultures from stem-derived calli of *H. perforatum* to enhance flavonoid production. By optimizing culture conditions and employing methyl jasmonate elicitation, the flavonoid content in these cultures reached up to 16 mg/g dry weight within a 20- to 25-day cultivation period (Wang et al., 2015). Furthermore, *Echinacea angustifolia*, a North American native species containing valuable bioactive compounds including quercetin derivatives and rutin glycosides, has emerged as another successful candidate for commercial-scale phytochemical production through advanced cell culture techniques (Gubser et al., 2021). These breakthroughs not only pave the way for a steady, high-quality supply of these bioactive compounds but also reduce the ecological impact associated with harvesting wild populations. An overview of the plant cell factory workflow is illustrated in **Figure 2**.

In summary, plant cell factories offer a scalable and environmentally friendly alternative to traditional methods, providing consistent flavonoid production for applications in pharmaceuticals, nutraceuticals, and other industries. Continued research into optimizing these cultures will be key to realizing their industrial potential.

### 5.3 Optimization of growth conditions and elicitors for enhanced flavonoid biosynthesis

Optimizing growth conditions can effectively enhance flavonoid production. Several factors, including light, temperature, and nutrient availability, significantly affect flavonoid biosynthesis. For instance, UV-B radiation affects the accumulation of secondary metabolites in plants. Interestingly, UV-B radiation rapidly triggers flavonoid biosynthesis, with adaptive responses occurring within minutes to hours (Shi and Liu, 2021). Temperature modulates enzymatic activity in secondary metabolite pathways, directly influencing flavonoid biosynthesis and accumulation. Both elevated and reduced temperatures impart substantial effects, with the latter being especially favorable for the production of flavonoids. In tea plants, where the dominant secondary metabolic pathway is flavonoid biosynthesis, an increase in temperature and light intensity boosts flavonoid production (Zhang et al., 2017). Anthocyanins, a significant class of flavonoids, are also induced by low temperatures. On the other hand, exposure to low temperatures leads to increased flavonoid accumulation in *Arabidopsis thaliana* (Bhatia et al., 2018). In maize (Zea mays L.), lowering the temperature from 23°C to 18°C increased leaf anthocyanin content by 35-fold (Pietrini and Massacci, 1998). The promoter of the R2R3 MYB transcription factor Ruby1 in blood orange (Citrus sinensis) contains a reverse transcriptional transposon that regulates low-temperature-induced anthocyanin accumulation. CsERF054 and CsERF061 bind to the DRE/CRT cis-element within this transposon, activating CsRuby1 transcription and anthocyanin synthesis. Furthermore, CsERF061 interacts with CsRuby1, forming a protein complex that synergistically activates anthocyanin synthesis genes (Wang et al., 2024). Nutrient availability significantly influences flavonoid biosynthesis. During the early growth stages, the synthesis and secretion of key substances in the flavonoid metabolic pathway vary with different nitrogen sources. Notably, nitrogen deficiency conditions lead to increased flavonoid synthesis and secretion (Li et al., 2024). Recent research on *Arabidopsis* has revealed that the brassinosteroid (BR)-responsive transcription factor BZR1 plays a critical role in enhancing anthocyanin biosynthesis under low nitrogen conditions. BZR1 directly binds to the promoter regions of PAP1/2, leading to increased transcriptional activity. Additionally, the physical interaction between BZR1 and PAP1/2 further amplifies this effect, thereby promoting anthocyanin biosynthesis. This dual mechanism underscores the importance of BZR1 in regulating anthocyanin production, which is crucial for plant stress responses and pigmentation (Lee et al., 2024).

Biotic elicitors, derived from organisms such as bacteria, fungi, algae, and polysaccharides, significantly influence flavonoid production (Bhaskar et al., 2022). For instance, bacterial elicitors like *Rhizobium rhizogenes* and *Escherichia coli* have been shown to increase genistein production by 94% and diosgenin by 9.1-fold, respectively. Recent research has focused on hormone-regulated flavonoid metabolic pathways. Salicylic acid (SA) alters the gene expression of key enzymes in secondary plant metabolism, boosting the production of bioactive compounds, including essential oils, phenolic acids, flavonoids, tannins, and alkaloids (Pacheco and Gorni, 2021). In *Dendrobium officinale*, methyl jasmonate (MeJA) upregulates MYC transcription factors—key regulators of flavonoid and jasmonate biosynthesis—resulting in enhanced accumulation of anthocyanins, rutin, hypericin, and isoquercitrin (Jia et al., 2024).

### 5.4 Application of artificial intelligence in metabolic engineering of plant cells

AI can play a crucial role in unraveling the intricate metabolic pathways within plant cells (Sears et al., 2024; Zampieri et al., 2019). These pathways govern the synthesis of vital compounds, including secondary metabolites (e.g., flavonoids), phytohormones, and bioactive molecules. AI algorithms enable the analysis of extensive datasets, prediction of enzyme functions, and identification of potential bottlenecks within these pathways (Shah et al., 2021). For instance, AI and machine learning (ML) techniques have been developed to predict enzyme functions and metabolic pathway memberships in *Arabidopsis thaliana*, enhancing our understanding of plant metabolism (Bai et al., 2024). Another case study is the ARCTICA framework, which integrates machine learning with metabolic modeling to simulate and control metabolic fluxes in cyanobacteria, providing a blueprint for similar applications in plant systems (Kugler and Stensjö, 2024). Gene-editing technologies, including CRISPR-Cas9, benefit from AI-driven target prediction, which pinpoints candidate genes involved in specific pathways. This precision allows for targeted genetic modifications, such as gene knockouts or overexpression, to fine-tune metabolic fluxes (Lin and Wong, 2018; Wada et al., 2020).

**Figure 3:**
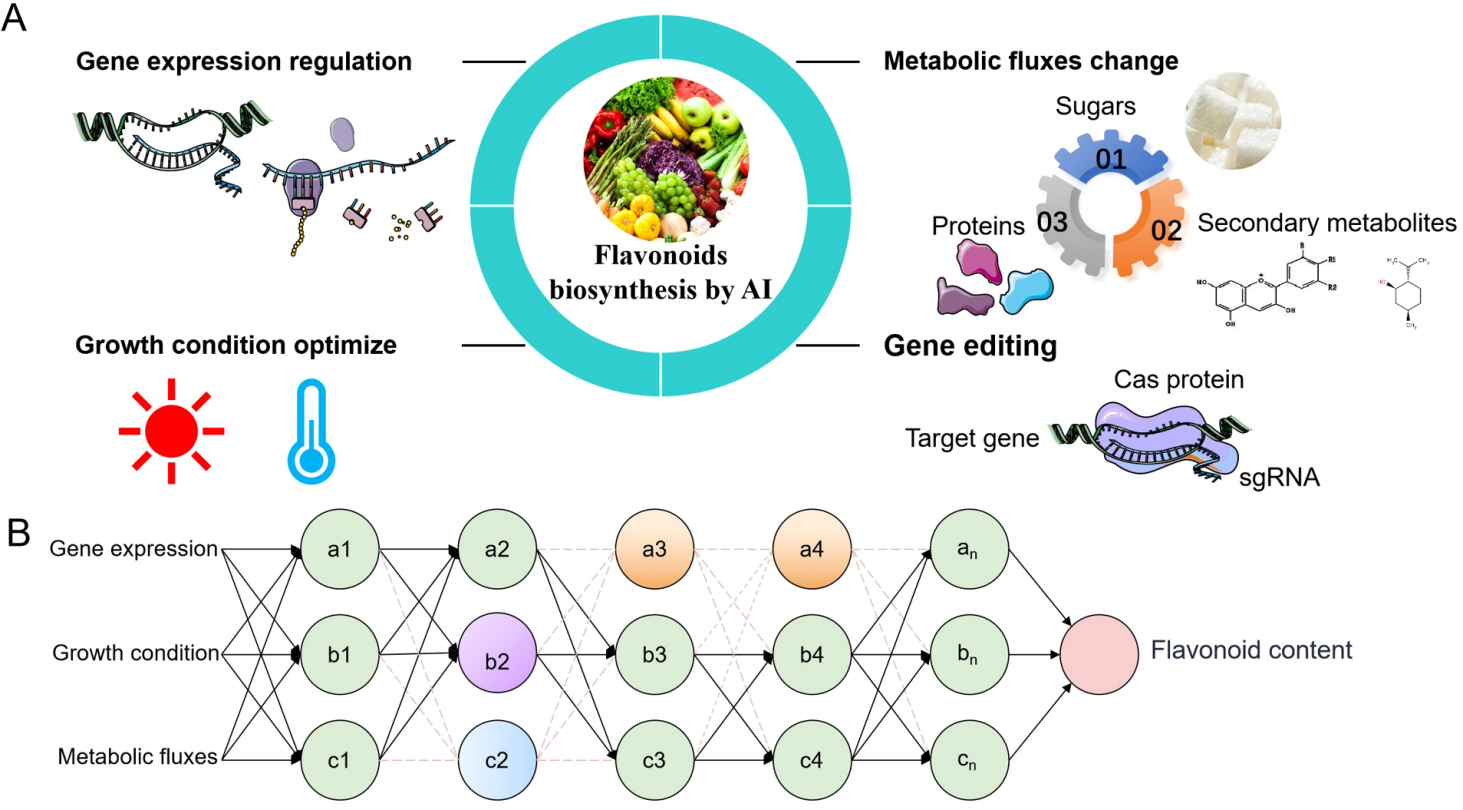
The potential role of artificial intelligence (AI) in promoting the biogenesis of flavonoids **(A)** AI enhances flavonoid production by modifying key elements of flavonoid biosynthesis. By altering the expression of genes related to flavonoid metabolism through AI design, adjusting the growth environment of plants or cells, redirecting plant metabolic flow, and editing target genes using CRISPR technology, the physiological state of plants can be optimized to produce target flavonoid metabolites under ideal conditions. **(B)** Briefly explain how artificial intelligence can promote flavonoid biosynthesis in three elements. (1) Expression of Core Genes: AI can modulate the expression of structural genes and regulatory factors involved in flavonoid synthesis, such as the PAL gene and various transcription factors (TFs). (2) Growth Conditions: AI can optimize growth conditions, including light intensity, temperature, and other environmental factors, to enhance flavonoid production. (3) Metabolic Flow Direction: AI can influence the direction of metabolic flow by increasing the rate of primary metabolite conversion to target flavonoids and reducing the production of other secondary metabolites.

In this review, we systematically evaluate the transformative potential of artificial intelligence (AI) in deciphering and engineering plant flavonoid metabolism (**Figure 3**). Modern AI frameworks, such as hybrid deep learning architectures and generative adversarial networks (GANs) enable *in silico* simulation of gene expression dynamics and their cascading effects on metabolic fluxes (Cheng et al., 2023). For instance, AI models trained on multi-omics datasets can predict how perturbations in transcriptional regulators (e.g., MYB, WRKY, or bZIP transcription factors) alter flavonoid biosynthetic pathways, thereby replacing conventional trial-and-error approaches with precision-guided experimental designs. Optimizing growth conditions, nutrient availability, and light exposure can further maximize flavonoid yields. AI’s integration into plant biology research extends beyond data analysis and prediction. AI facilitates the discovery of novel metabolic pathways and regulatory networks, offering insights into previously unexplored areas of plant metabolism. Advanced machine learning techniques, such as deep learning and neural networks, can identify patterns and correlations within complex biological data, leading to the identification of new targets for genetic manipulation. Furthermore, AI-driven approaches can predict the effects of environmental changes on plant metabolism, enabling researchers to develop strategies for improving crop resilience and productivity under varying conditions.

## 6 Conclusion

Compared with the primary metabolites, the amounts of secondary metabolites in plant cells are very low. However, flavonoid metabolites are the most abundant secondary metabolites in plant cells. Flavonoids have important physiological functions in plants. Since flavonoids themselves have antioxidant functions, they can regulate the signal pathway of plant reactive oxygen species and inhibit the production of excessive reactive oxygen species in plants, thereby relieving the damage of excessive reactive oxygen species to plants. In addition, flavonoids are involved in a variety of plant life activities, such as auxin transport, root and stem development, and regulation of plant response to external stress. Flavonoids have important roles not only in plants, but also in the treatment of diseases, such as cancer, Alzheimer’s disease, and atherosclerosis. Their strong antioxidant activity and free radical scavenging ability demonstrate therapeutic potential in preventing coronary heart disease, protecting liver function, reducing inflammation, and suppressing cancer progression. In addition, flavonoids are also widely used in several industries such as food, pharmaceutical and nutrition sectors, such as preservatives, pigments, antioxidants, etc. However, due to the inherently low biosynthesis of flavonoids in plant cells, industrial production currently relies on labor-intensive extraction from vast quantities of plant biomass, significantly hindering their widespread application in pharmaceuticals and nutraceuticals.

The biosynthesis of flavonoids has been the focus of research on plant secondary metabolism in recent decades. At the molecular level, the MYB transcription factor, bHLH transcription factor and WD40 protein form a MBW complex, which is the core regulatory element to regulate the expression of downstream flavonoid synthesis genes. The biosynthesis of downstream flavonoids can be precisely controlled by modulating the expression or composition of the MBW transcriptional complex. Furthermore, epigenetic mechanisms—including DNA methylation, histone acetylation, histone protein variants, and siRNA—exert dual regulatory effects: (1) indirectly influencing flavonoid synthesis by altering the expression of pathway-related genes through the MBW complex, and (2) directly regulating structural genes (e.g., CHS, FLS) that encode rate-limiting enzymes in the flavonoid biosynthetic pathway. This hierarchical regulatory network enables dynamic control of flavonoid production. In addition, the synthesis of flavonoids is strictly regulated by the external environment. External environmental factors such as light, temperature, CO_2_ content and moisture content also regulate the regulation of flavonoids at any time.

Recent advancements in genetic engineering techniques, particularly transgenic modification and precision genome editing, have revolutionized flavonoid biosynthesis in biological systems. These technologies enable both the introduction of heterologous biosynthetic pathways and fine-tuning of endogenous gene expression, resulting in substantial yield improvements of these valuable secondary metabolites. To address tissue-specific limitations in flavonoid accumulation, industrial applications increasingly employ optimized cell line selection strategies. This methodological shift not only enhances production stability but also facilitates more sustainable extraction processes through improved biomass utilization. The integration of artificial intelligence in metabolic engineering further expands the potential biosources for flavonoid production. Machine learning algorithms are accelerating the identification of non-traditional cellular platforms, moving beyond conventional plant-based systems. This technological convergence empowers researchers to tailor flavonoid biosynthesis according to therapeutic requirements while simultaneously enabling coproduction of related pharmacologically active compounds. Such developments suggest a paradigm shift in natural product manufacturing, where bioengineered systems may surpass traditional botanical sources in both efficiency and chemical diversity.
